# Seizure-Related Injuries among People with Epilepsy at the Outpatient Department of the University of Gondar Hospital, Northwest Ethiopia: Cross-Sectional Institutional-Based Study

**DOI:** 10.1155/2017/4970691

**Published:** 2017-12-11

**Authors:** Berhanu Boru Bifftu, Bewket Tadesse Tiruneh, Mengistu Mekonnen Kelkay, Nestanet Habte Bayu, Abebe Woldesellassie Tewolde, Wubet Worku Takele, Mehammed Adem Getnet, Abere Woretaw Azagew

**Affiliations:** Department of Nursing, University of Gondar College of Medicine and Health Science, Gondar, Ethiopia

## Abstract

**Background:**

The characteristics of epilepsy such as the episodic nature of impairment of consciousness and motor control, psychomotor comorbidity, seizure frequency, and side effects of antiepileptic drugs impact negatively on the physical safety of the patients. Physical injuries such as burn, fracture, dental loss, and hemorrhage affect the quality of patients' life to the extent of death. Thus, the main purpose of this study was to assess the prevalence of physical injury and associated factors among people with epilepsy.

**Methods:**

The study was carried out among 409 people with epilepsy. Cross-sectional study design was utilized to enroll the selected study participants using systematic random sampling technique. Binary and multivariable logistic regression were fitted to identify associated factors using an odds ratio and 95% CI.

**Results:**

The overall estimated prevalence of seizure-related physical injury was found to be 27.9%. Of the 27.9% seizure-related physical injuries, 12.5% had abrasions, 5.9% had burns, 4.4% had dental injuries, 2.2% had fractures, and 1.5% had head injuries and dislocations, respectively. Employment, 2-3 years duration of illness, seizure frequencies, and frequencies of drug taken were factors associated with physical injury.

**Conclusion:**

More than a quarter of the study participants experienced physical injury. Designing/strengthening injury prevention strategies is suggested especially for those who had uncontrolled seizure frequency for longer period of time.

## 1. Background

Epilepsy is the world's most common neurological disorder, affecting approximately 69 million people worldwide, majority of whom (90%) live in resource poor countries [1, 2]. In Ethiopia, epilepsy is a huge problem, with an estimated prevalence of 5.2 to 29.5/1000 people in large scale, rural and community based studies [2, 3]. Epilepsy affects the individuals' physical [4] and psychosocial life [1, 2, 5, 6].

Globally, most studies focus on the psychosocial impact of epilepsy, with little attention given to injury especially in developing countries like Ethiopia. Seizure characteristics such as the powerful muscular contractions, consisting of violent, convulsive movements that involve involuntary salivation, gnashing of teeth, and incontinence make people with epilepsy prone to physical injuries. The associated stigma leads the patients to live in isolated environment. In addition struggling with challenges like chilly colds, rain, heat, and harassments also increases the risk of injuries [4, 6–13]. Some of the causes of injuries include falling down onto open fire and getting burnt during epileptic seizure, falling down onto hard surfaces like stones and other objects leading to physical harm, and physical assault such as being stabbed.

Physical injuries are common among people with epilepsy compared to the general population with an estimated prevalence of 0.6% to 47.3% [4, 8, 9, 13]. The incidence of physical injuries has significant impact on the life of people with epilepsy (PWE) (such as psychopathology, intracranial hemorrhage, and fracture) worsening epilepsy outcomes and even leading to mortality [4, 7–10, 14]. The anticipation and risk of injuries are also associated with other negative outcomes such as inactivity, isolation, and dependency [4].

In Africa, including Ethiopia, however the impact of epilepsy on physical injury is also not well documented, and the limited available evidence revealed that the occurrences of physical injury negatively impact on the life of PWE with a high mortality rate compared to general population [2, 15–17]. For example in Ethiopia, two-year follow-up of death rate revealed 31.6/1000 for people with epilepsy compared to 16.4/1000 for general population [2]. Another study also revealed 9% serious physical injuries [16]. Thus, the main purpose of this study was to assess the type, frequency, and associated factors of seizure-related physical injury among people with epilepsy at the University of Gondar Hospital, Northwest Ethiopia.

## 2. Methods

### 2.1. Study Design, Area, and Period

Institutional-based cross-sectional quantitative study design was conducted at the University of Gondar Hospital (UoGH), from November 1/2015 to December 30/2015. University of Gondar Hospital is located in Gondar town, 748 kilometers from the capital city of Ethiopia, Addis Ababa.

### 2.2. Participants, Sample Size Determination, and Sampling Procedure

Participants were confirmed individuals with clinical diagnosis of epilepsy having follow-up care at the outpatient department of the University of Gondar Hospital. Single population proportion formula (with 5% margin of error, 95% confidence level, and 50% proportion) was used to calculate the sample size for the study and was found to be 422 (including 10% nonresponse rate). Confirmed individuals with a clinical diagnosis of epilepsy coming for follow-up with an age greater than or equal to 18 years for the last 12 months were taken from patient records to get the interval of participants per day. A systematic random sampling technique was employed to select the participants.

### 2.3. Data Collection Instruments

Data were collected by interviewing participants using an Amharic version of semistructured questionnaires and by reviewing patient charts. The questionnaires include sociodemographic and clinical factors such as age at the onset of the illness, duration of illness, seizure frequencies, frequencies and number of drug taken per day, and poly-pharmacy. For the assessments of physical injuries, authors developed semistructured questionnaires through literature reviews of similar studies [8–10, 12, 16]. The presence of self-reported physical injury was assessed by using the statement “in the past 12 months, have you experienced any seizure-related injury?” with response options of “Yes” or “No” and for those who responded “Yes,” they are asked about (a) type of injury using the predefined options (i) submersion, (ii) burn, (iii) fracture, (iv) head injury, (v) soft-tissue injury, (vi) dental injury and accident, and (vii) others specified with the possibilities of multiple responses and (b) frequencies of injuries. In this study, seizure-related physical injury was defined as one or more of any injury (abrasion, burn, dental injury, fracture and head injury, and dislocation) resulting from seizure. (b) Frequencies of injuries: seizure frequency was assessed by asking “how many times?” For the assessment of family social support, a single item with predefined options utilized (i.e., “how do you rate your social support (immediate family-parent, sibling, and child)?”; possible responses to this question were (a) Low, (b) Moderate, and (c) High). Furthermore, the current name of drug used and number and frequency of drug taken per day were also obtained from patient chart.

### 2.4. Data Analysis

The completed questionnaires were coded and entered into EPI info version 3.5.3 statistical software and then exported to SPSS windows version 20 program for analysis. Descriptive statistics (frequencies, tables, percentages, means, and standard deviation) was used for sociodemographic and clinical factors and seizure-related physical injury. Bivariate and multivariable logistic regressions with odds ratio of 95% confidence interval (CI) were used to assess the association between the seizures-related physical injuries and independent variables. All factors with a *p* value < 0.05 were accepted as a cut-off point. Details of the methodology have been described [18].

## 3. Ethical Consideration

The study proposal was approved by the Ethical Review Board of The University of Gondar. A formal letter of permission was also obtained from the hospital and submitted to the epilepsy clinic. The necessary information about the study was given to the participants and for those participants who agreed to participate in the study a written informed consent was obtained and they were interviewed in private room.

## 4. Results

A Total of 409 people with epilepsy participated in this study with a response rate of 96.9%.

### 4.1. Sociodemographic Characteristics

Majority of participants were males 257 (63%). The mean (SD) age of the participants was 28.63 ± 9.70 years. About half of the participants 199 (48.7%) completed primary education, 375 (91.7%) were Orthodox Christians, and 247 (60.5%) were married. Three hundred and eighty-eight (94.9%) of the participants were of Amhara ethnic group. Out of 409 participant, 240 (58.7%) were self-employed and 218 (53.3%) were living in urban areas (Table 1). Overall, 113 (27.6%) of the participants seizure onset was within age ranged from 18 to 24 years. Of all participants, 144 (35.2%) had seizure duration of 2–5 years, 293 (71.6%) had seizure frequencies of 1–11 per year, and 195 (47.7%) of the participants were taking their drugs twice per day. Regarding the perceived severity of the epilepsy, 167 (40.8%) of the participants perceived epilepsy as highly severe. A detailed description of sociodemographic and clinical features has been published previously [18].

### 4.2. Prevalence, Type, and Frequencies of Physical Injury

The overall estimated prevalence of seizure-related physical injury was found to be 27.9%. Of this 27.9% of seizure-related physical injuries, 12.5% had abrasions, 5.9% had burns, 4.4% had dental injuries, 2.2% had fractures, and 1.5% had head injuries and dislocations, respectively. Regarding the frequencies of injuries, 72 (17.6%) of the participants experienced once, 33 (8.1%) of the participants experienced twice, and 3 (0.3%) of the participants experienced thrice.

### 4.3. Factors Associated with Physical Injury

From the bivariate analysis: age, employment status, residence, family social support, onset of the illness, duration of the illness, perceived severity of the epilepsy, seizure frequencies, and frequencies of drug taken per day were factors associated with seizure-related physical injury. Factors with *p* value < 0.2 were entered in to the multivariable analysis. From the multivariable analysis, those who are unemployed (AOR = 0.80, CI: 0.026, 0.971), with 2–5 years of illness duration (AOR = 3.24, CI: (1.190, 8.839)), seizure frequencies [1–11/years (AOR = 1.80, CI: 1.089, 9.221), ≥1/month (AOR = 3.61, CI: 2.390, 8.854)], and frequencies of drug taken per day (AOR = 1.81, CI: 1.072, 9.425) were factors associated with physical injury at *p* value < 0.05 (Table 1).

## 5. Discussion

The main findings of this study revealed that physical injuries occurred in more than one-fourth of people with epilepsy. Those who were unemployed, urban residence, severity of the illness, duration of illness, and frequencies of drug taken per day were associated with seizure-related physical injuries. The overall estimated prevalence of seizure-related physical injury and type of injury is similar with a study carried out in UK (27%); the study reported that soft-tissue injury accounts for 61%, burns account for 17%, head injuries account for 14%, and orthopaedic injuries account for 5% [19]. The reported prevalence of seizure-related physical injuries in our study is lower than the studies carried out in Thailand 38.6% [20], Nigeria 45.6% to 67.8% [21, 22], and Iran 47.3% [12]. However, the findings from our study were higher than the previous studies carried out in Ethiopia (9%) [16], in a European cohort 17% [10], and Nova Scotia (17%) [23]. These differences may be due to the seizure severity, duration of the seizure, and type of the illness. Different frequencies and patterns of injuries have been reported in studies, but the most commonly reported injuries were fractures, burns, motor vehicle accidents, dental injury, and soft-tissue injury [8, 10, 21, 23, 24]. These evidences are supported by the finding of our study. From this it is possible to observe the impact of seizure-related physical injuries on the safety of people with epilepsy.

Compared to governmental workers those participants with unemployed status were twenty percent times (AOR = 0.80, CI: 0.026, 0.971) less likely to experience physical injuries. This may be due to the nature of work our study participants were involved in because different work or professions have different risk of injury. Studies have revealed that epilepsy does not usually force employers into taking extra safety precautions in the workplace as far as they are not involved in some restricted job such as driving and other works that have flashing or flickering lights till the seizure is controlled for at least a year [25, 26].

Those patients with duration of illness ranging from 2 to 5 years were more than three times (AOR = 3.24, CI: (1.190, 8.839)) likely to experience physical injuries compared to those patients with duration of illness less than one year. This is consistent with other studies reporting that early onset of symptom is a risk factor for the occurrence of injuries [12, 23, 24]. This may be due to seizures with its intended risk of injuries, which is consistent with other studies [23, 24].

Those patients who took their drugs three times per day were around two times more likely to experience seizure-related physical injuries compared to those patient who took their AEDs once per day. This may be due to the cumulative effects of drug side effects such as sedation, vomiting, constipation, joint pain, and others affecting patients' self-control. This finding has been supported by other studies [12, 21, 24, 25]. The other possible explanation could be also the severity of the epilepsy (people taking drugs three times per day are more probably affected by more severe epilepsies).

## 6. Limitations of the Study

Recall and response biases might have occurred, since we relied on the respondents' retrospective recall of the last 12 months of information. Because of stigma and discrimination attached to the illness, participants may hide the true information. Lack of similar studies in Ethiopia limited the comparison of the study. Despite these limitations, the findings of our study should inform and help all the stakeholders to take corrective action.

## 7. Conclusion and Recommendations

More than a quarter of the study participants experienced physical injury. Those who were unemployed, 2–5 years of illness duration, seizure frequencies ≥1/month and frequencies of drugs taken per day were factors associated with seizure-related physical injuries. Thus, we suggested the need for designing/strengthening injury prevention strategies such as safe working environment with special attention for those who have uncontrolled seizure for longer duration of time with close observation for those taking drugs frequently.

## Figures and Tables

**Table 1:** Factors associated with physical injury (bivariate and multivariable) analysis (*n* = 409).

Explanatory variables	Physical injury		COR (95% CI)	AOR (95% CI)	*p*value
	Yes *N* (%)	No *N* (%)			
Age					
18–24	54 (32.5)	112 (67.5)	4.34 (0.166, 7.893)		
25–34	36 (28.6)	90 (71.4)	3.60 (0.029, 9.172)		
35–44	21 (24.1)	66 (75.9)	2.86 (0.296, 6.004)		
≥45	3 (10)	27 (90)	1		
Employment					
Government	6 (20.7)	23 (79.3)	1	1	
Private	511 (21.2)	189 (78.8)	10.50 (6.302, 18.201)	2.32 (0.117, 6.983)	0.408
Students	21 (31.8)	45 (68.2)	1.79 (0.198, 5.327)	5.10 (0.086, 10.955)	0.094
Unemployed	36 (48.6)	38 (51.4)	3.63 (1.101, 8.897)	0.80 (0.026, 0.971)	0.007
Residential					
Rural	42 (22)	149 (78)	0.57 (0.023, 0.661)		
Urban	72 (33)	146 (67)	1		
Social support					
High	51 (20.5)	198 (79.5)	0.36 (0.103, 2.096)		
Moderate	36 (37.6)	59 (62.1)	0.70.89 (0.995, 7.331)		
Low	27 (41.5)	38 (58.5)	1		
Age at onset of epilepsy in year					
<18	66 (38)	108 (62)	4.07 (1.167, 9.038)		
18–24	24 (21.2)	89 (78.8)	1.80 (1.438, 9.211)		
25–34	18 (23.7)	58 (76.3)	2.07 (1.103, 5.223)		
≥35	6 (13)	40 (87)	1		
Duration of the illness in year					
<1	9 (22)	32 (78)	1	1	
2–5	24 (16.7)	120 (83.3)	0.71 (0.011, 0.962)	3.24 (1.190, 8.839)	<0.001
6–10	45 (18.1)	73 (61.9)	2.19 (1.001, 7.033)	5.08 (0.113, 6.372)	0.700
≥11	36 (34)	70 (66)	1.83 (0.011, 8.662)	9.52 (0.165, 3.970)	0.092
Seizure frequency per year					
0	21 (24.7)	64 (75.3)	1	1	
1–11/year	75 (25.6)	218 (74.4)	1.05 (1.001, 7.033)	1.80 (1.089, 9.221)	0.003
≥1/month	18 (58.1)	13 (41.9)	4.22 (0.011, 8.662)	3.61 (2.390, 8.854)	<0.001
Therapy (number of AEDs)					
Monotherapy	72 (23.3)	237 (76.7)	1		
Poly-therapy	42 (42)	58 (58)	4.32 (0.011, 8.662)		
Frequencies of AEDs taken per day					
Once	30 (17.2)	144 (82.8)	1	1	
Twice	60 (30.8)	135 (69.2)	2.13 (1.001, 7.033)	2.30 (0.099, 11.522)	
Thrice	24 (60)	16 (40)	7.20 (0.011, 8.662)	1.81 (1.072, 9.425)	
